# Titin: roles in cardiac function and diseases

**DOI:** 10.3389/fphys.2024.1385821

**Published:** 2024-04-10

**Authors:** Dawson Stroik, Zachery R. Gregorich, Farhan Raza, Ying Ge, Wei Guo

**Affiliations:** ^1^ Cellular and Molecular Pathology Program, Department of Pathology and Laboratory Medicine, School of Medicine and Public Health, University of Wisconsin-Madison, Madison, WI, United States; ^2^ Department of Animal and Dairy Sciences, College of Agriculture and Life Science, University of Wisconsin-Madison, Madison, WI, United States; ^3^ Department of Medicine, School of Medicine and Public Health, University of Wisconsin-Madison, Madison, WI, United States; ^4^ Department of Cell and Regenerative Biology, School of Medicine and Public Health, University of Wisconsin-Madison, Madison, WI, United States

**Keywords:** titin, passive stiffness, dilated cardiomyopathy (DCM), heart failure with preserved ejection fraction (HFpEF), diabetic cardiomyopathy (DbCM)

## Abstract

The giant protein titin is an essential component of muscle sarcomeres. A single titin molecule spans half a sarcomere and mediates diverse functions along its length by virtue of its unique domains. The A-band of titin functions as a molecular blueprint that defines the length of the thick filaments, the I-band constitutes a molecular spring that determines cell-based passive stiffness, and various domains, including the Z-disk, I-band, and M-line, serve as scaffolds for stretch-sensing signaling pathways that mediate mechanotransduction. This review aims to discuss recent insights into titin’s functional roles and their relationship to cardiac function. The role of titin in heart diseases, such as dilated cardiomyopathy and heart failure with preserved ejection fraction, as well as its potential as a therapeutic target, is also discussed.

## 1 Introduction

Titin (also known as connectin) constitutes the so-called third filament component of muscle sarcomeres (Maruyama, 1976; Wang et al., 1979). With a molecular weight of ∼3–4 MDa (in humans), titin is the largest protein expressed in mammals, extending from the Z-disk to the M-band where the N- and C-termini are anchored, respectively. Titin is divided into four portions based on the orientation of the molecule within the half sarcomere. These portions, which include the Z-disk, A-band, I-band, and M-line, determine titin’s functional roles. Titin’s A-band serves as a molecular blueprint that determines the length of the thick filament and helps to properly orient it within the sarcomere (Whiting et al., 1989; Tonino et al., 2017; Bennett et al., 2020). Titin functions as a molecular spring that gives rise to passive tension mainly through three extensible segments located in the I-band: the tandem immunoglobulin (Ig)-like domain, the repeating PEVK [proline (P), glutamate (E), valine (V) and lysine (K)] domain, and N2B domain (Labeit and Kolmerer, 1995). In addition to these segments, the binding of chaperones to the N2A domain also regulates its spring-like properties directly through binding to this domain and indirectly via modulation of N2A phosphorylation (Lun et al., 2014; Lanzicher et al., 2020). These elastic segments function as a spring that supports early diastolic recoil and late diastolic resistance to stretch (Granzier and Labeit, 2002; Granzier and Labeit, 2004; Hamdani and Paulus, 2013; Guo and Sun, 2018). In effect, titin generates a stretch-resisting force that functions to restore sarcomere resting or “slack” length (Linke et al., 1996). Several different mechanisms regulate titin-based passive stiffness, chief among which is alterative splicing in the I-band portion of titin (Forbes et al., 2005). In the heart, splicing in this portion of the molecule produces two cardiac-specific N2BA and N2B (arise from alternative splicing) titin isoforms that have been studied in great depth and will be the focus of this review (Zou et al., 2015; Chen et al., 2018). Splicing has also been shown to produce smaller Novex-1-3 isoforms while the Cronos isoform is produced from a unique promoter (Bang et al., 2001; Zou et al., 2015). However, the function(s) of these isoforms in cardiac structure and function, particularly in cardiomyopathy, is presently unclear. Titin also serves as a molecular signaling mediator that activates stretch-sensitive signaling pathways to reshape the myocardium in response to sarcomere stress through protein complexes tethered in its Z-disk, I-band, and M-line portions (Granzier and Labeit, 2002; Granzier and Labeit, 2004; Granzier and Labeit, 2006; Linke, 2008; Linke and Krüger, 2010; Hamdani and Paulus, 2013; Linke and Hamdani, 2014; Guo and Sun, 2018). The aim of this review is to discuss the structure and functions of titin’s various domains, as well as how titin splicing and post-translational modifications (PTMs) contribute to these functions. We also discuss heart diseases in which titin plays a role, such as dilated cardiomyopathy (DCM), heart failure with preserved ejection fraction (HFpEF), and diabetic cardiomyopathy (DbCM) (Herman et al., 2012; Zile et al., 2015; Hopf et al., 2018). Lastly, we discuss the potential therapeutic approaches for the treatment of heart diseases by targeting titin.

## 2 Structure and localization of cardiac titin in the sarcomere

**FIGURE 1:**
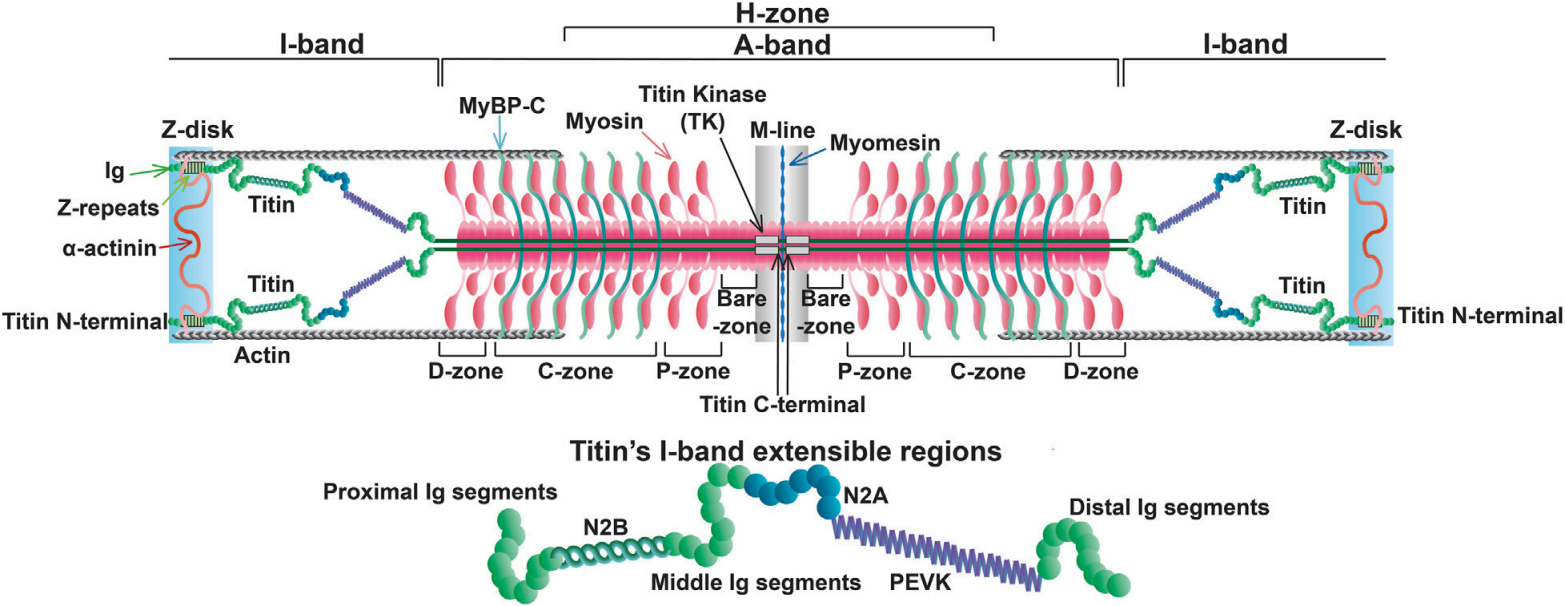
Sarcomere structure at slack length with full length titin.

The titin gene (*TTN*) is located on chromosome 2q31 in humans and encompasses 364 exons (a single non-coding exon followed by 363 coding exons) (Bang et al., 2001)*.* This paper will only refer to the 363 coding exons. One titin protein molecule spans one-half of a sarcomere, transversing the Z-disk, I-band, A-band, and M-line with its N- and C-terminal portions anchored in the Z-disk and M-line, respectively (Figure 1) (Wang et al., 1979).

**FIGURE 2:**
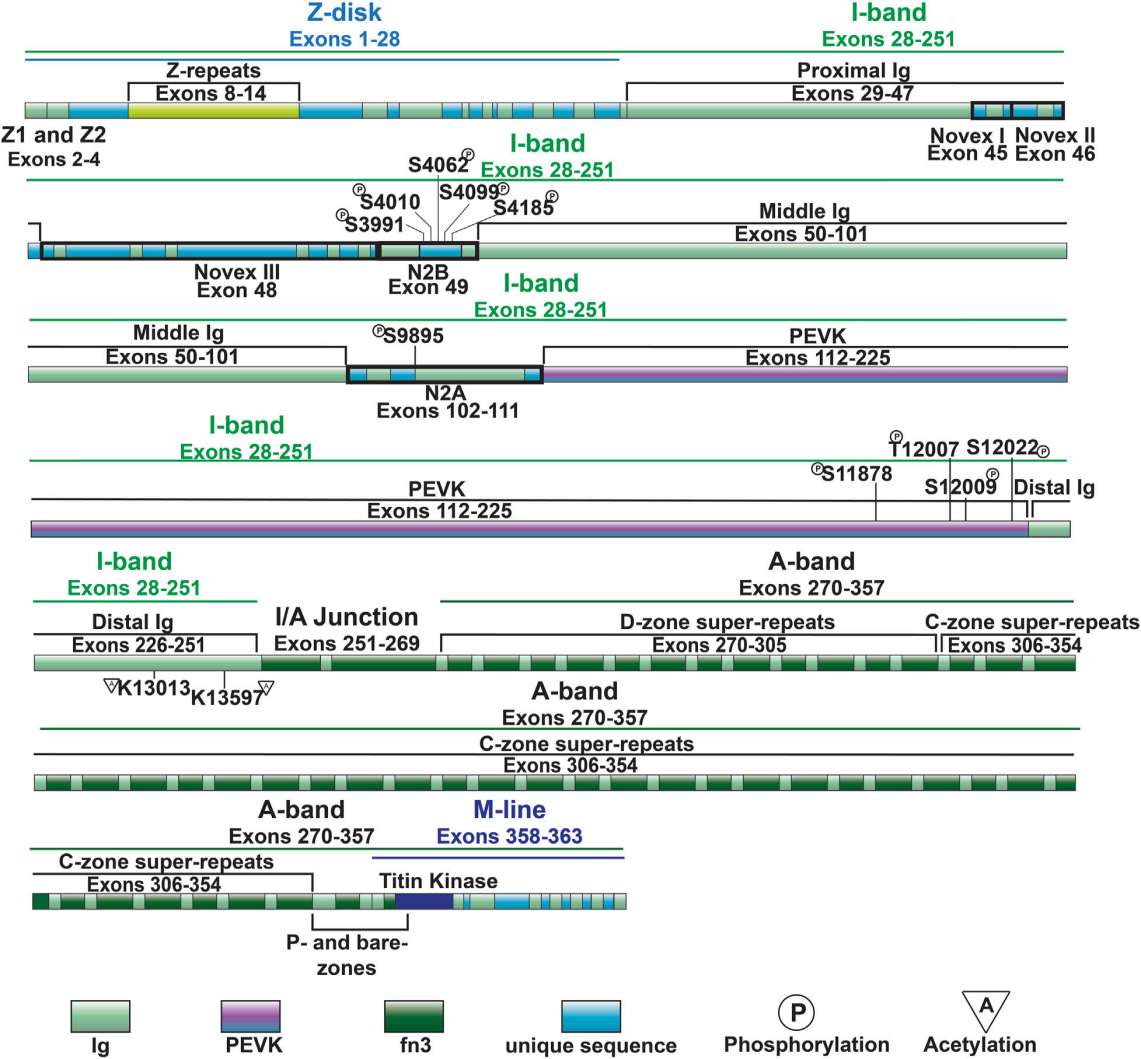
Full-length titin domain map with exons, sites of common I-band PTMs and A-band super-repeats noted.

Z-disk and Z-disk/I-band junction of titin is encoded by coding exons 1–28 and anchors the N-terminus of the protein in the Z-disk. This portion of the molecule consists primarily of consecutive immunoglobulin-like (Ig) domains and 45-residue repeat motifs termed Z-repeats (Figures 1, 2) (Gautel et al., 1996). The Ig-like domains are made of eight β-strands arranged in two sheets packed face-to-face making their structure similar to traditional Ig domains (Pfuhl and Pastore, 1995; Improta et al., 1996). The first and second Z-disk Ig-like domains (Z1 and Z2) in titin interact with telethonin (also known as T-cap) (Gregorio et al., 1998), which crosslinks titin molecules from adjacent sarcomeres in an anti-parallel arrangement (Zou et al., 2006). In addition, the Z-repeats have been shown to mediate interactions with the C-terminal domain of the actin-crosslinking protein α-actinin (Ohtsuka et al., 1997; Sorimachi et al., 1997; Joseph et al., 2001) (Figure 1). The interaction between titin and α-actinin provides strong anchoring of titin within the Z-disk through multiple Z-repeat/α-actinin bonds (Grison et al., 2017). Recent work has added to this list of Z-disk titin-interacting proteins, although many of these interactions remain to be validated and their functional significance is unclear (Rudolph et al., 2020; Filomena et al., 2021).

I-band titin represents the extensible portion of the molecule and is encoded by exons 28–251, with the I/A-band junction encoded by exons 251–269 (Labeit and Kolmerer, 1995). The I-band portion of titin can be separated into six distinct regions: the proximal Ig-like domain (exons 29–47), cardiac-specific N2B segment (exon 49), middle Ig segment (exons 50–101), N2A segment (exons 102–111), PEVK region (exons 112–225), and distal Ig-like domain (exons 226–251) (Figures 1, 2) (Labeit and Kolmerer, 1995). As the names indicate, the proximal, middle, and distal Ig-like domains encode series of tandemly arranged Ig-like domains (Labeit and Kolmerer, 1995). On the other hand, the N2B and N2A segments are comprised of three and four Ig-like domains, respectively, each with additional unique sequences (572 amino acids in human N2B and 104 amino acids in human N2A) (Labeit and Kolmerer, 1995).

**FIGURE 3:**
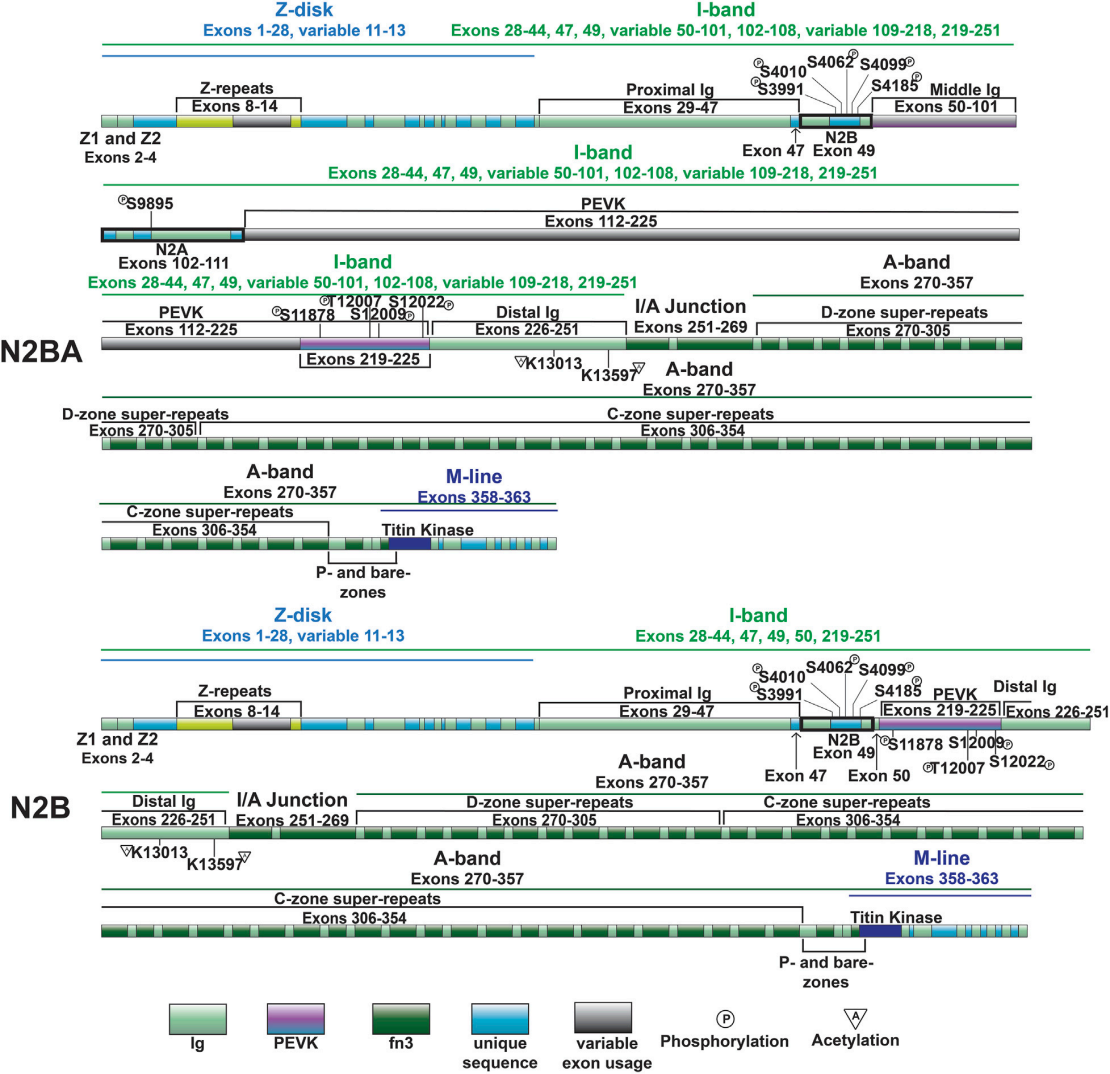
Domain map of cardiac titin isoforms—N2BA and N2B.

Titin’s A-band (encoded by exons 270–357) uniquely contains fibronectin-type III-like (fn3) domains that account for approximately 70% of the A-band portion (Figure 2) (Labeit et al., 1990; Labeit and Kolmerer, 1995). Together with the Ig-like domains, the fn3 domains form two distinct types of super-repeats that define two disparate zones in the A-band: the distal (D)- and central (C)-zones (Labeit and Kolmerer, 1995). The I/A-band junction and P-zone (exons 355–358) also contain fn3 domains, however, this zone does not follow the super-repeat structure found in the D- and C-zones (Labeit et al., 1997). The D-zone co-localizes with the tips of the myosin filaments at the border of the A-band and is composed of 6 repeats of Ig-fn3-fn3-Ig-fn3-fn3-fn3 (Figures 2, 3) (Labeit and Kolmerer, 1995). On the other hand, the C-zone has the pattern Ig-fn3-fn3-Ig-fn3-fn3-fn3-Ig-fn3-fn3-fn3 repeated 11 times and extends from the C-zone to the bare zone (Figures 2, 3) (Labeit and Kolmerer, 1995). The fn3 domains in titin consist of seven β-strands comprising two sheets forming a β-sandwich structure (Goll et al., 1998). It has been shown that the fn3 domain sequences in the A-band can be separated into three distinct fn3 sequence types, the arrangement of which defines distinct repeat units in the D- and C-zones, respectively (Fleming et al., 2023). Critically, these super-repeats serve as a template that regulates the length of the thick filament (Tonino et al., 2017), which will be discussed in greater detail in a later section.

M-line titin is encoded by exons 358–363 (known as Mex1-Mex6) and tethers the C-terminus of the protein to the M-line (Kolmerer et al., 1996). In the M-line, titin molecules from adjacent half sarcomeres overlap, forming a continuous filament that stabilizes the sarcomere (Figure 1). A large proportion of cardiac titin expresses all six exons, Mex1-Mex6, unlike skeletal muscle, which often lacks Mex5 (Kolmerer et al., 1996). If Mex5 is not expressed in cardiac tissue, pathogenic phenotypes often develop (Marcello et al., 2022). Mex 1 encodes the titin kinase (TK), which is responsible for binding multiple different proteins (Kolmerer et al., 1996; Puchner et al., 2008). The role of these M-line regions change throughout development, and the absence of these exons results in embryonic lethality (Gotthardt et al., 2003). Under mechanical load, sarcomeres initially assemble but fail to grow laterally in the absence of a continuous titin filament and eventually disassemble, underscoring the important structural function of this portion in the beating heart (Mayans et al., 1998).

## 3 Functional roles of titin in cardiac muscle

Titin is a functionally pleiotropic protein with specific functions mediated by its unique regions. Proper sarcomere formation requires titin, as myosin uses it as a blueprint that determines the length of the thick filament (Tonino et al., 2017). Titin also contains spring-like domains that grant it elasticity. These elastic properties allow titin to act as a molecular spring that contributes significantly to the passive stiffness of the myocardium (Gautel and Goulding, 1996; Linke et al., 1996; Tskhovrebova and Trinick, 1997; Linke et al., 1998; Trombitás et al., 1998). Titin also plays a role in mechanosignaling by serving as a scaffold for stretch-sensing protein complexes located in the Z-disk, I-band, and M-line (Linke and Krüger, 2010; Kötter et al., 2014a; van der Pijl et al., 2021). These functions will be discussed in greater detail below.

### 3.1 Function as a molecular blueprint

Early study of purified myosin assembled into “synthetic thick filaments” revealed that these filaments lacked the homogeneous length exhibited by thick filaments purified from muscle (HUXLEY, 1963). Observations such as this raised the question of how thick filament length is regulated *in vivo*. A critical finding came in 1989 when it was shown that the length of the large super-repeats in the C-zone of A-band titin matched the ∼43 nm spacing of myosin heads in the corresponding portion of the thick filament (Fürst et al., 1989). This finding, together with data providing evidence of interactions between titin and myosin (Murayama et al., 1989), lead to the hypothesis that titin determines the length of thick filaments (Whiting et al., 1989). Although this hypothesis remained contentious for many years, more recent evidence has confirmed that this is, in fact, the case (Tonino et al., 2017). Tonino et al. (2017) generated a novel mouse model in which the first two super-repeats of the C-zone were deleted. Structural studies confirmed that the length of the sarcomeric A-band was reduced in both cardiac and skeletal muscles from these mice (Tonino et al., 2017). Further support has been provided by analysis of the axial disposition of titin on the thick filament using super-resolution microscopy (Bennett et al., 2020). Thus, it can be concluded that A-band titin serves as a molecular blueprint for the sarcomeric A-band, thereby controlling the length of the thick filaments.

### 3.2 Function as a molecular spring

Passive stiffness/tension is the resistance to stretching that muscles exhibit when not actively contracting. In the heart, passive stiffness is determined by a variety of factors, including the cytoskeletal filaments, extracellular matrix (e.g., collagen), and titin, with the latter two contributing the greatest in the working range of cardiac sarcomeres (Linke et al., 1994; Granzier and Irving, 1995; Loescher et al., 2023). In particular, it has been shown that in the working range of cardiac sarcomeres (∼1.9–2.2 µm) titin-based stiffness predominates in the lower end of the working range and collagen-based stiffness at the higher end and beyond (Granzier and Irving, 1995). Titin-based stiffness arises from the reversible straightening of extensible domains in titin’s I-band (Gautel and Goulding, 1996; Linke et al., 1996; Tskhovrebova and Trinick, 1997; Linke et al., 1998; Trombitás et al., 1998). These extensible domains include the Ig and PEVK domains, as well as the N2B and N2A domains (Figure 1).

#### 3.2.1 Spring-like domain: Ig-like domains

When the sarcomeres are at slack length, titin’s I-band exists in a folded conformation with the tandem Ig-like domains in a contracted configuration (Trombitás et al., 1995; Granzier et al., 1996). When stretched, the connecting segments between Ig-like domains straighten first, followed by extension of the PEVK domain (Gautel and Goulding, 1996; Linke et al., 1996; Tskhovrebova and Trinick, 1997; Linke et al., 1998; Trombitás et al., 1998) and N2B unique sequence (N2Bus) (Trombitás et al., 1999). Extension of the tandem Ig-like domains contributes to passive stiffness at the lower end of the working range of cardiac sarcomeres (<2.0 µm) (Trombitás et al., 1999). An early mechanism proposed to explain titin elasticity posited that the Ig-like domains themselves unfold, as was observed in isolated titin molecules (Erickson, 1994; Lu et al., 1998). A series of papers published in the late 1990s suggested that such unfolding *in vivo* would require non-physiological levels of force (Kellermayer et al., 1997; Rief et al., 1997; Tskhovrebova et al., 1997) and, thus, for many years it was thought that unfolding of the Ig-like domains did not occur naturally. However, the findings of a recent study suggest that Ig-like domain unfolding may occur at physiological sarcomere lengths and forces of 6–8 pN (Rivas-Pardo et al., 2016). These findings not only again raise the possibility that Ig-like domain unfolding occurs, but also put forward that refolding of these domains actively contributes to contraction (Rivas-Pardo et al., 2016). Additional studies will be necessary to confirm these findings.

The major mechanism regulating titin-based stiffness is alternative splicing of exons encoding Ig-like domains in the middle Ig segment (discussed in greater detail below) (Freiburg et al., 2000; Guo et al., 2010), however it has been found that the extensibility of domains such as the Ig-like domains can also be regulated by PTMs. For example, it has been shown that cryptic (i.e., buried) cysteine residues in the Ig-like domains can undergo reversible S-glutathionylation, which decreases titin-based passive stiffness by hindering refolding of these domains following stretch (Alegre-Cebollada et al., 2014; Loescher et al., 2020). Several CaMKII phosphorylation sites have been identified within Ig-like domains and the linker sequences between such domains in the I-band (Hamdani et al., 2013a). It has been proposed that phosphorylation of these sites may affect passive stiffness through a similar mechanism to oxidation (Hamdani et al., 2017), although whether this is the case remains to be determined. Besides PTMs, protein-protein interactions involving titin’s Ig-like domains have also been found to impact titin-based stiffness. Under stretching conditions, heat shock protein (HSP) 27 and αB-crystallin can bind to Ig-like domains and decrease passive stiffness (Kötter et al., 2014b). This interaction is believed to occur only upon unfolding of the Ig-like domains as HSP27 and αB-crystallin rarely localized to Ig-like domains in the longer and more compliant N2BA titin isoform, which consists of Ig-like domains that are less likely to unfold (Kötter et al., 2014b).

#### 3.2.2 Spring-like domain: PEVK

The PEVK region of titin is encoded by exons 112–225 in the I-band and is highly extensible (Gautel and Goulding, 1996; Linke et al., 1996). Extension of the PEVK is a major determinant of passive stiffness at cardiac sarcomere lengths greater than 2.0 µm (Trombitás et al., 1999). The elasticity of the PEVK domain is due, at least in part, to this domain acting as an entropic string (Linke et al., 2002). However, there are also charge contributions that impact stiffness, meaning that the spring-like feature of the region is not entirely entropically driven (Forbes et al., 2005). If regions of the PEVK are treated with an ionic solution, persistence length increases while Debye–Hückel length decreases, providing evidence that electrostatic interactions in this region also play an important part in determining the stiffness of the PEVK domain (Forbes et al., 2005).

**FIGURE 4:**
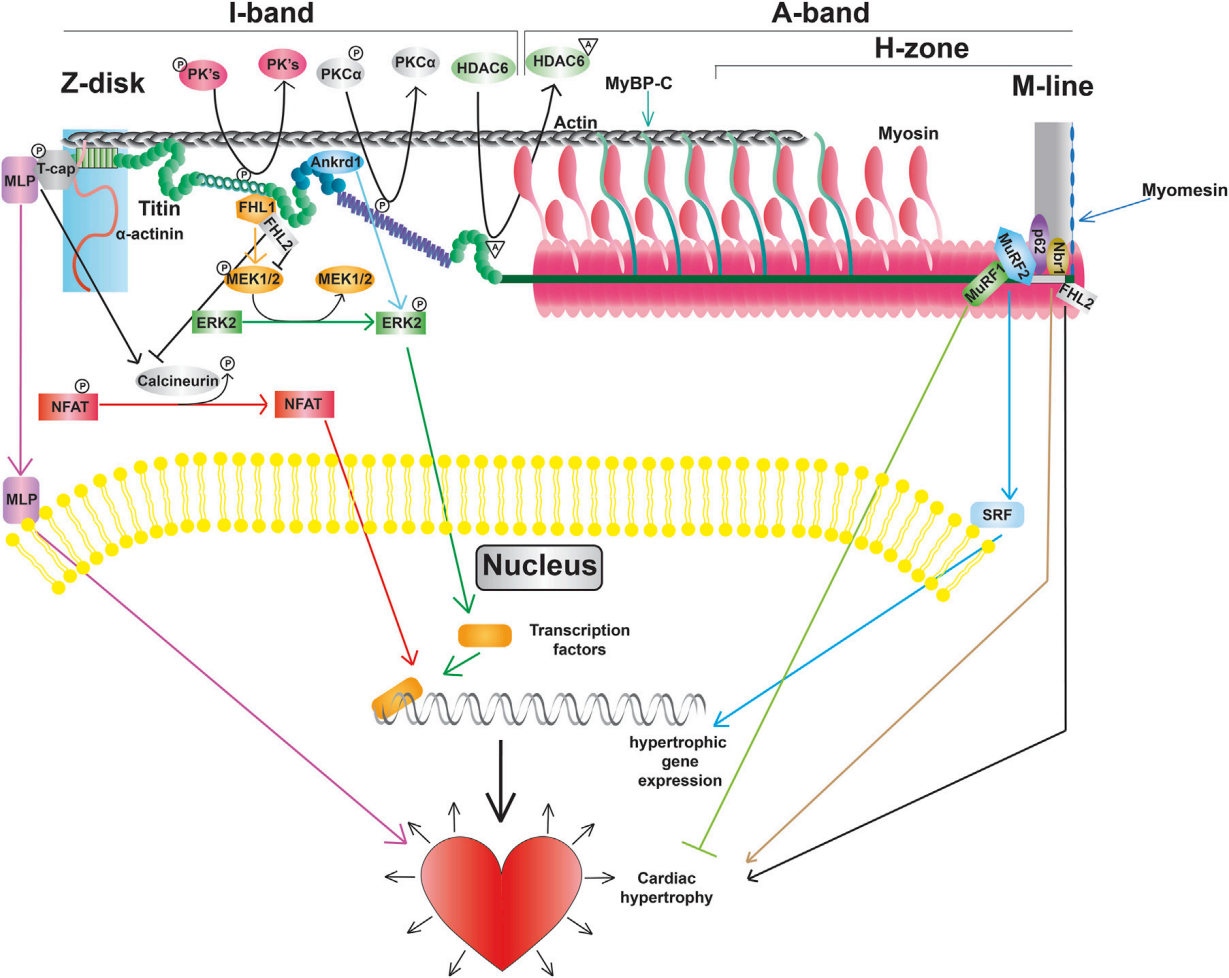
Titin modifications and signaling pathways, including promotion of transcription factors, altered gene expression, and cardiac hypertrophy.

Similar to the Ig-like domains of the middle Ig segment, control of PEVK stiffness can be regulated by alternative splicing of the exons encoding this domain (discussed below) (Freiburg et al., 2000; Guo et al., 2010), as well as through PTMs. Phosphorylation sites in the PEVK region of human titin include S11878 and S12022, which can both be phosphorylated by PKCα (Hidalgo et al., 2009) (Figure 4). PKCα-mediated phosphorylation of these sites increases passive tension (Hidalgo et al., 2009). Phosphorylation of additional residues in the PEVK domain, including T12869, S12871, and S12884 in mouse [corresponding to T12007, S12009, and S12022 in human titin, respectively (Figures 2, 3)], by CaMKII isoforms has also been described (Hamdani et al., 2013a). Intriguingly, treatment of single skinned mouse cardiomyocytes isolated from CaMKIIδ/γ double knockout mice with CaMKIIδ decreased titin-based passive stiffness (Hamdani et al., 2013a). Additional sites of CaMKII phosphorylation were also identified in other parts of the I-band, such as the N2Bus (Hamdani et al., 2013a). Given that the experimental design did not permit for the relative contributions of PEVK and N2B phosphorylation to passive stiffness to be assessed (Hamdani et al., 2013a), it is unclear whether the decrease was due to a dominant effect of N2B phosphorylation (discussed in the next section) or if the modulation of PEVK-associated titin stiffness is site-dependent.

Recent evidence indicates that acetylation of residues within the PEVK segment also modulates passive stiffness (Lin et al., 2022). Initially it was found that myofibril resting tension was elevated in mice deficient in histone deacetylase 6 (HDAC6) (Lin et al., 2022). Importantly, decreased titin stiffness following incubation with recombinant HDAC6 did not occur in isolated myofibril preparations from mice lacking 282 amino acids of the PEVK/Ig-like region of titin, indicating that acetylation of this region is responsible for acetylation-associated modulation of titin stiffness (Lin et al., 2022). Subsequently, mass spectrometry identified K13013 and K13597 as sites of acetylation downstream of the PEVK domain (Lin et al., 2022). *Hdac6* deficient mice develop greater diastolic dysfunction in response to hypertension and aging, suggesting that HDAC6-mediated control of titin phosphorylation could be important for normal cardiac function and in response to stress and normal aging (Lin et al., 2022).

#### 3.2.3 Spring-like domains: N2B and N2A

The cardiac specific N2B domain is encoded by exon 49 and contains three Ig-like domains and a 572 amino acid sequence unique to the domain (Figure 3) (Labeit and Kolmerer, 1995). Studies have shown the N2Bus contributes to passive stiffness via unfolding near the upper end of the physiological cardiac sarcomere length range (∼2.2 µm) (Helmes et al., 1999; Trombitás et al., 1999). The importance of N2B domain extensibility is underscored by the finding that deletion of the domain in mice leads to diastolic dysfunction secondary to increased extension of the remaining extensible elements (tandem Ig and PEVK domains) and increased titin stiffness (Radke et al., 2007). The N2A domain of the N2BA cardiac titin isoform (also expressed in the skeletal muscle N2A titin isoform) is another region of titin with elastic properties, characterized by four Ig-like domains and a 104 amino acid unique sequence (Labeit and Kolmerer, 1995). This domains unique α-helices surrounded by disordered region allow it to act as an entropic spring like the N2B domain (Lanzicher et al., 2020; van der Pijl et al., 2021).

The N2Bus is a well-known target for a variety of kinases. Common phosphorylation sites in the N2Bus include S3991, S4010, S4062, S4099, and S4185, among others (Figure 3). Each site can be phosphorylated by multiple different protein kinases such as PKA (Yamasaki et al., 2002), PKG (Krüger et al., 2009), PKD (Herwig et al., 2020), ERK2 (Perkin et al., 2015), and/or CaMKII (Hamdani et al., 2013a; Perkin et al., 2015) and dephosphorylated by the serine/threonine phosphotase PP5 (Krysiak et al., 2018). Phosphorylation of N2Bus by these different kinases decreases titin-based stiffness while dephosphorylation increases it (Yamasaki et al., 2002; Krüger et al., 2009; Perkin et al., 2015; Krysiak et al., 2018; Herwig et al., 2020). Although the mechanism whereby the phosphorylation of sites in the N2Bus produces a decrease in passive stiffness remains to be determined, it has been postulated that the introduction of a negatively charged group to the already negatively charged N2Bus improves extensibility by reducing the force required to extend this segment through increased intramolecular electrostatic repulsion (Koser et al., 2019). The N2A domain is also able to be phosphorylated by PKA at S9895, however it does not appear to contribute much, if at all, to titin’s passive stiffness. Instead, phosphorylation of the region helps mediate its protein interactions, which will be discussed in further detail in Section 3.4.2 (Lanzicher et al., 2020).

Stiffness of the N2Bus is also regulated by oxidative modifications. For example, it has been shown that disulfide bridges may form between cysteine residues in the N2Bus (Grützner et al., 2009). Although direct evidence for the formation of these disulfide bonds in the heart is lacking, treatment of isolated human heart myofibrils with the reducing agent thioredoxin reduced titin-based stiffness to a degree consistent with increased extensibility of the N2Bus (Grützner et al., 2009), providing circumstantial evidence for their existence.

### 3.3 Control of I-band extensibility through alternative splicing

As mentioned above, the most well-studied mechanism regulating titin extensibility involves alternative splicing in titin’s I-band region (Freiburg et al., 2000; Guo et al., 2010). The inclusion or exclusion of many exons in the middle Ig and PEVK domains dramatically affects the length of titin and, thus, its extensibility. The heart expresses two major classes of titin isoforms: a smaller and stiffer N2B isoform and larger more compliant N2BA isoforms (Figure 3) (Cazorla et al., 2000; Freiburg et al., 2000). The N2B isoform is stiffer due to exclusion of a greater number of exons in the middle Ig and PEVK domains while N2BA isoforms have greater extensibility because of inclusion of these exons and correspondingly longer Ig and PEVK domains. In a healthy adult human heart, the N2BA isoform comprises 30%–50% of titin proteins, while the N2B isoform makes up 50%–70% of titin proteins (Cazorla et al., 2000; Neagoe et al., 2002). The expression of cardiac titin isoforms has been shown to change during development and in pathological conditions (Bell et al., 2000; Neagoe et al., 2002; Wu et al., 2002; Warren et al., 2003; Makarenko et al., 2004; Nagueh et al., 2004; Opitz et al., 2004; Warren et al., 2004; Shapiro et al., 2007; Borbély et al., 2009; Hudson et al., 2011; Hamdani et al., 2013b; Zhu et al., 2017).

In 2012, we identified deletion of the gene encoding RNA binding motif protein 20 (RBM20) as the cause of altered titin splicing in rats deficient in titin splicing (Guo et al., 2012). This discovery made RBM20 the first factor known to regulate titin size through alternative splicing. In a follow up study, we determined that RBM20-mediated control of titin size occurs predominantly via exon exclusion although this was exon-dependent (Li et al., 2013). When present, RBM20 promotes exclusion of exons between 51 and 218 encoding the middle Ig and PEVK segments, thereby favoring the production of N2B titin (Figure 3) (Li et al., 2013). On the other hand, at reduced levels, or when RBM20 is absent, inclusion of these exons is favored, leading to the production of more compliant N2BA titin isoforms with longer middle Ig and PEVK segments (Figure 3) (Guo et al., 2012; Li et al., 2013). In the absence of RBM20, a giant N2BA isoform of titin is expressed (Borbély et al., 2009). Mechanistically, RBM20 promotes exon skipping by binding to sites downstream of target exons (Dauksaite and Gotthardt, 2018), although the precise mechanism has not been determined.

A growing list of additional splicing factors have been identified that can also regulate titin splicing in the heart. These factors include RBM24 (Liu et al., 2019), PTB4 (Dauksaite and Gotthardt, 2018), SLM2 (Boeckel et al., 2022), and RBPMS (Gan et al., 2023). Importantly, studies have only provided evidence for the regulation of select exons in titin for each of these factors and, apart from RBPMS (Gan et al., 2023), none of these factors have been shown to significantly switch titin size (Dauksaite and Gotthardt, 2018; Liu et al., 2019; Boeckel et al., 2022). RBPMS is involved in the inclusion of the N2B exon and loss of this protein in mice produced an apparent decrease in the size of both N2B and N2BA titin isoforms (Gan et al., 2023). This size change produces titin isoforms similar in size to those produced via N2B deletion (Gan et al., 2023). Other confirmed exon skipping events associated with loss of RBPMS were restricted to exons outside the I-band (Gan et al., 2023), which are generally constitutively expressed in cardiac titin. Thus, although exclusion of these exons produced a DCM-like phenotype in *Rbpms* deficient mice, the physiological significance of RBPMS-regulated titin splicing in the healthy heart is unclear at the present time. Therefore, RBM20 remains the major factor regulating alternative splicing of the *TTN* gene in health and disease.

### 3.4 Function as a mediator of mechanical signaling

Beyond titin’s role as a blueprint for the sarcomeric A-band and as a molecular spring responsible for myocardial passive stiffness, it also has an important role as an integrator and transducer of mechanical signals (Linke and Krüger, 2010; Kötter et al., 2014a; van der Pijl et al., 2021). Titin serves as a scaffold for a variety of mechano-sensitive signaling complexes localized in the Z-disk, I-band, and M-line. In addition, titin is also a substrate for signaling pathways enabling the refinement of passive stiffness through PTMs in regions such as the N2Bus and PEVK segment as discussed above (Figure 4).

#### 3.4.1 Z-disk signaling

The Z-disk of titin serves as a hub for pro-hypertrophic signaling pathways. Hypertrophic signaling at the Z-disk is regulated by the stress sensor MLP, which has been shown to bind to the titin-T-cap complex (Knöll et al., 2002). Study of mice lacking a single copy of the Mlp gene revealed that MLP plays an important role in localization of the phosphatase calcineurin to the Z-disk (Heineke et al., 2005). Moreover, transcriptional activation of NFAT was blunted in these mice following myocardial infarction demonstrating that the Z-disk-localized MLP-calcineurin complex is required for the stress-induced activation of the pro-hypertrophic calcineurin-NFAT signaling pathway (Heineke et al., 2005). In addition to activation of calcineurin-NFAT signaling, MLP itself can also translocate to the nucleus (Boateng et al., 2007). The finding that treatment with cell-permeable synthetic peptides containing the putative nuclear localization signal of MLP blocked not only nuclear translocation of the protein, but also increased protein accumulation in response to phenylephrine, suggests that nuclear shuttling of MLP is also important for adaptation to hypertrophic stimuli (Boateng et al., 2009). Interestingly, MLP and Ankrd1 together can transport PKCα to intercalated discs, which is often seen in DCM, and deletion of both proteins restores proper PKCα localization and function (Lange et al., 2016).

#### 3.4.2 I-band signaling

Beyond determining extensibility, titin’s I-band segments also serve as docking sites for protein complexes that mediate critical signaling events. The spring-like N2B domain interacts with FHL1, FHL2, and FHL3 (four and a half LIM protein 1, 2, and 3, respectively) (Lange et al., 2002; Sheikh et al., 2008). Interestingly, FHL1-mediated signaling has been shown to promote the activation of the pro-hypertrophic MAPK signaling cascade (Sheikh et al., 2008), while FHL2 opposes the hypertrophic response (Figure 4) (Kong et al., 2001). The functional role of FHL3 in cardiac muscle is not well understood, however, it has been shown to localize to titin’s N2B region and expression is severely deregulated in cardiomyopathies (Puchner et al., 2008). In addition to FHL proteins, I-band titin has also been shown to interact with proteins belonging to the muscle ankyrin repeat protein (MARP) family (Miller et al., 2003). One member of the MARP family, ANKRD1 (also known as CARP), has been implicated in cardiac hypertrophy due to upregulation during cardiogenesis and in response to hypertrophy stimuli (Kuo et al., 1999; Aihara et al., 2000). Similar to MLP, mechanical stimulation promotes translocation of ANKRD1 from the sarcomeres (where it co-localizes with the N2A domain of titin) to the nucleus (Miller et al., 2003). While a number of I-band protein interactors and signaling pathways are consistent between cardiac and skeletal muscle titin, there are some proteins, such as calpain-3, that only interact with the skeletal muscle titin N2A domain (Ojima et al., 2014).

#### 3.4.3 M-line signaling

A portion of M-line titin encodes a kinase, TK, that is located near the A-band/M-line junction (Labeit et al., 1992). Evidence from early studies suggested that TK plays an important role during muscle development (Mayans et al., 1998). Additional data suggested that this kinase could also be activated in response to mechanical strain making it a putative stress sensor (Puchner et al., 2008). However, more recent studies indicated that TK is a pseudokinase (Puchner et al., 2008), making it unlikely that the kinase activity of this domain is required for signal transduction in response to strain. Nevertheless, there is evidence that a portion of this domain can undergo reversible unfolding in response to stretch (Puchner et al., 2008), and TK does serve as an important scaffold for a variety of signaling proteins. In particular, the autophagy receptor Nbr1 has been shown to recruit p62 and MuRF2 to the TK domain (Lange et al., 2005). The E3 ubiquitin ligase MuRF1 can also interact with a sequence near the TK domain (Centner et al., 2001). Bogomolovas et al. showed that MuRF1 can ubiquitinate the TK domain in a stretch-dependent manner and this ubiquitination is required for recruitment of Nbr1 and p62 (Bogomolovas et al., 2021). Another location for E3 ubiquitin ligase binding on titin is at the C-terminal portion of the A-band at Ig141/Ig142/Fn3-132 (A168-170), which can bind MURF1, 2, and 3 (Müller et al., 2021). These pathways are currently thought to be important for breakdown of the sarcomeres (Bogomolovas et al., 2021), although further investigation will be required to determine whether this is the case.

## 4 Titin and heart diseases

Due to titin’s myriad functional roles in the heart, it is not surprising that disruption of these functions by things such as mutations is associated with the development of heart diseases. Indeed, titin has been implicated in a variety of heart muscle diseases among which dilated cardiomyopathy (DCM) and heart failure with preserved ejection fraction (HFpEF)/diabetic cardiomyopathy (DbCM) are the most prominent (Table 1) (Herman et al., 2012; Zile et al., 2015; Hopf et al., 2018). In the following section we will briefly discuss the contribution of titin to these diseases and provide updates regarding mechanism based on recently published studies.

**TABLE 1:** Cardiac diseases associated with titin.

	TTNtv	Missense DCM	HFpEF	DbCM
Sources	22, 122–132	125, 133–135, 137–138	23, 141–146	23, 24, 74, 79, 93
Cardiac changes	Enlarged left ventricle with thin cardiac walls	Enlarged left ventricle with thin cardiac walls	Left ventricular hypertrophy and diastolic dysfunction	Left ventricular hypertrophy and diastolic dysfunction
Mutation location	Most commonly titin’s A-band or M-line	Titin C3575S—in I21 of I-band	None reported	None reported
Titin PTM changes	None reported	None reported	Decreased I- and A-band acetylation Increased PEVK phosphorylation	Decreased N2B phosphorylation Increased PEVK phosphorylation
Titin isoform changes	Increased exon usage	None reported	Favors N2B	Favors N2BA

### 4.1 Titin in DCM

Titin truncating variants (TTNtvs) represent the most common cause of familial and sporadic DCM, accounting for upwards of 25% of familial cases and ∼15% of sporadic cases (Herman et al., 2012; Pugh et al., 2014; Roberts et al., 2015; Haggerty et al., 2019; Mazzarotto et al., 2020). Surprisingly, TTNtvs are also found in ∼1–2% of the general population (Pugh et al., 2014; Roberts et al., 2015; Schafer et al., 2017). Pathogenic TTNtvs are typically carried in the heterozygous state and are overrepresented in the A-band (Herman et al., 2012; Pugh et al., 2014; Roberts et al., 2015). This phenomenon is thought to be because exons encoding the A-band are constitutively expressed (Roberts et al., 2015). Over the years, a variety of hypotheses have been proposed to explain the pathogenicity of TTNtvs; however, the two most popular have been haploinsufficiency and the poison peptide hypothesis (Yotti et al., 2019). In the case of the former, titin production from the single unaffected allele is insufficient to maintain appropriate cardiac function, while the latter posits that the truncated protein acts in a manner that disrupts normal heart function. The findings of recent studies indicate that both mechanisms contribute to the development of DCM in TTNtv carriers.

One of the primary issues that has hindered acceptance of the poison peptide hypothesis has been the inability to detect truncated titin proteins in the hearts of DCM patients with TTNtvs (Roberts et al., 2015; Vikhorev et al., 2017). However, using a combination of gel electrophoresis and antibodies against the N- and C-termini of titin, McAfee et al. (2021) were able to detect appropriately sized TTNtvs in heart samples from patients with DCM caused by TTNtvs. An independent study from the Linke lab published at the same time made similar findings using similar methods (Fomin et al., 2021). Additionally, TTNtv cannot be properly ubiquitinated for degradation due to the M-line portion of titin not being expressed and have been detected in insoluble granules containing TTNtv in human iPSC-CMs (Huang et al., 2023). In addition to the poison peptide theory, Fomin et al. (2021) detected a decrease in total titin expression that correlated with decreased left ventricular ejection fraction, indicating that haploinsufficiency also plays a role in disease severity. Interestingly, they also found no difference in the ratio of M-band versus Z-disk titin based on immunofluorescence staining (Fomin et al., 2021), suggesting that the truncated proteins, which would be labeled by the Z-disk but not M-line antibodies, are not incorporated into the sarcomeres. Conversely, truncated titin proteins precipitated with skinned myofibril preparations from human DCM hearts as determined by gel electrophoresis (McAfee et al., 2021; Kellermayer et al., 2024). Moreover, using super-resolution microscopy, Kellermayer et al. (2024) detected reduced titin signal based on immunofluorescence staining of titin with N- and C-terminal antibodies however when TTNtv does assemble within the sarcomere, there is a 49-fold increase in disassembly (Bogomolovas et al., 2021). Considering this conflicting data, the question of whether truncated titin proteins are incorporated into the sarcomeres or if they exist in intracellular aggregates (Fomin et al., 2021) requires further investigation.

In addition to TTNtvs, a long list of missense variants in titin have also been identified in association with DCM, as well as peripartum cardiomyopathy (PPCM) (Merlo et al., 2013; Pugh et al., 2014; van Spaendonck-Zwarts et al., 2014; Begay et al., 2015; Akinrinade et al., 2019); however, evidence indicating a causal association between these variants and disease has been largely lacking. Recently, Domínguez et al. (2023) identified a case of familial DCM in a Spanish family carrying substitution of a highly conserved cysteine at position 3892 to serine. Linkage analysis produced a family-specific 2-point logarithm of the odds score of 3.96, which strongly supports that the variant is related to the phenotype (Domínguez et al., 2023). While prior studies of families carrying TTNtvs have implicated titin missense variants in disease (Gerull et al., 2002), this study provides the strongest evidence to date that missense variants in titin can cause DCM. Although the mechanism will require further investigation, limited evidence from circular dichroism spectroscopy suggests that this variant may destabilize the protein at physiological temperatures potentially causing disease through haploinsufficiency (Domínguez et al., 2023). Other missense mutations in titin have been associated with PPCM in mothers during late pregnancy and early post-partum (van Spaendonck-Zwarts et al., 2014). While there are many phenotypic similarities between DCM and PPCM, they differ in disease onset and the fact that a proportion of PPCM patients are able to fully recover, while DCM can only be managed (van Spaendonck-Zwarts et al., 2014). The mechanisms leading to the development of PPCM are not fully understood, but there are many similarities with DCM. Prior studies have shown that N2BA titin is increased in PPCM, similar to what has been shown in DCM, however the impact on passive force/tension is contradictory in the literature (van Spaendonck-Zwarts et al., 2014; Bollen et al., 2017). Also impacting passive tension, PKA- and PKC-mediated phosphorylation of titin at S4010 and S12022 are both significantly reduced in PPCM patients, while S12022 is unaffected in DCM patients (Bollen et al., 2017). Another titin contribution to PPCM is that the increased oxidative stress caused by pregnancy also contributes to the pathogenic phenotype (Bollen et al., 2014).

### 4.2 Titin in HFpEF/DbCM

HFpEF and DbCM produce very similar cardiac phenotypes with cardiac hypertrophy, increased cardiac stiffness, and impaired diastolic function. Understanding the physiological mechanisms of HFpEF is one of the greatest unmet needs in cardiovascular disease due to a lack of effective treatments. Within 4 years of HFpEF diagnosis, the mortality rate is over 30% (Burkhoff, 2012). One of the biggest risk factors for HFpEF development is hypertension (Tam et al., 2017; Tadic et al., 2018). Granzier et al. showed that hypertensive mouse models favored the N2B titin isoform over the N2BA compared to control mice, which increases titin-based myocardial stiffness and decreases diastolic function (Hutchinson et al., 2015). As HFpEF develops, PTMs in titin’s elastic domains change to increase the overall titin-based myocardial stiffness. Patients with HFpEF often have increased PEVK phosphorylation at S11878 and decreased I- and A-band acetylation (Zile et al., 2015; Koser et al., 2022). There is also reduced N2B phosphorylation at S3991, S4080, and S4185 causing increased stiffness (see previous sections) (Zile et al., 2015; Koser et al., 2022). Further supporting altered titin PTMs, treatment of skinned cardiomyocytes isolated from a patient with HFpEF with PKA was able to restore passive force to near the healthy range (Borbély et al., 2005). Both titin isoform switching and altered PTMs play major roles in the increased titin-based myocardial stiffness found in HFpEF patients.

Like HFpEF, people suffering from DbCM often have impaired diastolic function because of altered PTMs in titin’s elastic I-band domains. Contrary to HFpEF, DbCM patients have the opposite effect in terms of titin isoform switching and promote N2BA expression (Hopf et al., 2018). The major alternative splicing factor RBM20 has increased expression in the presence of insulin, meaning that people who are insulin resistant have decreased RBM20 levels (Zhu et al., 2017). In turn, this promotes the expression of the N2BA titin isoform over N2B, decreasing titin-based myocardial stiffness. On the other hand, biopsies reveal those suffering from DbCM have altered N2B and I-band PTMs (Krüger et al., 2009; Zile et al., 2015; Lin et al., 2022). Patients with type 2 diabetes had decreased phosphorylation of S4099 (phosphorylated by PKG) in the N2B region and increased phosphorylation of S11878 (phosphorylated by PKCα) in the PEVK region (Krüger et al., 2009; Zile et al., 2015; Lin et al., 2022). The result of these altered PTMs is increased titin-based stiffness. Improving insulin sensitivity to modulate titin stiffness through targeting NRG-1 or via treatment with metformin reverses the altered PTMs of the N2B and PEVK domains found in diabetes patients, showing its therapeutic potential for diastolic dysfunction (Hopf et al., 2018).

## 5 Future perspectives: targeting titin in heart disease

Titin’s status as a causative agent in diseases such as DCM, as well as its role as an important modulator of myocardial passive stiffness (and by extension diastolic function), make it an appealing therapeutic target. In the case of DCM caused by variants in titin, two therapeutic strategies are currently being explored to target titin. The first is the use of antisense oligonucleotides (ASOs) to promote skipping of variant-containing exons has been explored as a potential strategy to treat pathogenic variants in titin that cause DCM (Gramlich et al., 2015). ASO-mediated strategies have shown promise in both mice and cultured cells (Gramlich et al., 2015; Hahn et al., 2019). Given that ASOs have already been approved for the treatment of other diseases (Crooke et al., 2021), such as Duchenne’s muscular dystrophy, ASO-based exon skipping strategies represent an accessible strategy to treat DCM caused by certain disruptive variants in titin. It has been predicted that upwards of 94 exons in titin may be suitable for therapeutic deletion to treat DCM-associated titin variants (Rodriguez-Polo and Behr, 2022). More recently, CRISPR-Cas9 genome editing was used to correct TTNtvs in human iPSC-CMs (Romano et al., 2022). Expansion of these results to model organisms such as mice will be an important step towards demonstrating the therapeutic potential of this strategy. In the case of idiopathic DCM, potential therapeutic approaches could involve targeting RBM20. Most people suffering from DCM have a shift in titin isoforms to favor the longer N2BA isoform paired with decreased ventricular passive stiffness and systolic dysfunction (LeWinter and Granzier, 2014). By increasing RBM20 expression through regulation of transcriptional factors, it would shift titin to favor the N2B isoform, restoring titin-based myocardial passive stiffness (Guo et al., 2012).

Diastolic dysfunction is a key element of heart diseases such as HFpEF and DbCM (Borlaug, 2014; Jia et al., 2016). Titin size changes resulting from alternative splicing plays a major role in myocardial stiffness, while PTMs in titin’s spring-like domain fine-tune myocardial stiffness, as discussed above (Forbes et al., 2005; Krüger et al., 2009; Raskin et al., 2012; Hamdani et al., 2013a; Hamdani et al., 2013b; Hidalgo et al., 2013; Kötter et al., 2013; Perkin et al., 2015; Krysiak et al., 2018; Herwig et al., 2020; Michel et al., 2020; Loescher et al., 2022). Thus, either manipulating titin size switching or altering PTMs in titin could reduce diastolic stiffness and, therefore, dysfunction. Given that RBM20 is the primary regulator of titin size through alternative splicing (Guo et al., 2012), this protein is an attractive target to modulate titin size and improve diastolic function. Reducing RBM20 expression through genetic editing or ASO-mediated RNA degradation has provided proof-of-concept evidence that increasing the expression of larger titin isoforms improves diastolic function in rodent models of HFpEF (Hutchinson et al., 2015; Hinze et al., 2016; Methawasin et al., 2016; Radke et al., 2021). It is important to point out that RBM20 also controls the splicing genes related to Ca^2+^-handling (Guo et al., 2012; Guo et al., 2018) and loss of RBM20-dependent splicing of these genes predisposes the heart to arrhythmia (Guo et al., 2012). Thus, targeting RBM20 expression to modulate titin size may have detrimental effects. The consensus sequence containing the core element UCUU has been shown to be the binding motif for RBM20 in titin mRNA (Maatz et al., 2014). Therefore, an alternative therapeutic option would be to design ASOs targeting the RBM20 binding sites on the titin pre-mRNA. Theoretically, such a strategy could reduce diastolic stiffness in HFpEF/DbCM by promoting exon inclusion and the expression of larger titin isoforms without affecting the splicing of other RBM20 target genes. Another option would be to combine both approaches. RBM20 binds many locations on titin and so designing ASO’s to inhibit RBM20 binding to titin mRNA poses difficulties. By reducing RBM20 expression, the titin ASO’s may lead to a greater increase in N2BA titin isoform expression. Nevertheless, the reduction in RBM20 expression must be highly regulated to prevent severe alterations in RBM20-mediated alternative splicing of other mRNA targets such as Ca^2+^-handling genes (Guo et al., 2012).

In addition to titin isoform switching, the modulation of titin-based stiffness through PTMs represents another potential treatment strategy to reduce diastolic stiffness in HFpEF and DbCM or increased diastolic stiffness in DCM. Studies show that patients with DbCM often display cardiac stiffening despite increased expression of more compliant N2BA titin (van Heerebeek et al., 2008). The potential mechanisms could be the increased phosphorylation in N2Bus and decreased phosphorylation in PEVK region (Yamasaki et al., 2002; Hidalgo et al., 2009; Krüger et al., 2009; Perkin et al., 2015; Krysiak et al., 2018; Herwig et al., 2020). Examination of titin phosphorylation in the hearts of diabetic patients revealed reduced phosphorylation of S4099 (in N2Bus) and increased phosphorylation of S11878 (in PEVK) that can account for increased passive stiffness (Hopf et al., 2018). Treating diabetic rats with NRG-1 (drug that improves glucose tolerance) restored diastolic function and titin phosphorylation to similar levels to wildtype through PKG- and PKCα-mediated phosphorylation (Hopf et al., 2018). NRG-1 treated rats also had an improvement in PKA-mediated phosphorylation, however not to the same extent (Hopf et al., 2018). Interestingly, diabetic rats treated with metformin (increases insulin sensitivity) have increased phosphorylation of titin S11878 in the PEVK (Hopf et al., 2018). It is noteworthy that there is conflicting data in the literature regarding the effect of metformin treatment on titin PTMs as others have shown little to no effect on PEVK phosphorylation but increased phosphorylation of PKA sites in the N2Bus (Slater et al., 2019). Studies also reveal that histone deacetylase 6 (HDAC6) can deacetylate K13013 and K13597 near the PEVK region (Figure 4) (Lin et al., 2022). Lack of HDAC6 or treated with HDAC6 inhibitors in mice increases myocardial stiffness (Lin et al., 2022). In addition to HDAC6, study found that deacetylase SIRT1 can reduce stiffness in rat cardiomyocytes (Abdellatif et al., 2021). Overall, these studies show that targeting PTMs in titin represents another therapeutic option to reduce diastolic stiffness in HFpEF/DbCM. Modulating titin stiffness through PTM’s may also be valuable as a supplement for other treatments. For example, regulation of titin PTMs and favored production of more compliant N2BA titin isoforms through ASOs could be used to synergistically decrease titin-based stiffeness in diseases with diastolic dysfunction, such as in DbCM. Therefore, it would be logical to conclude that regulating titin PTM’s could be especially beneficial in combination with therapies that also increase titin N2BA expression.
